# Supplementary material for: Recognition and Chaperoning by Pex19, Followed by Trafficking and Membrane Insertion of the Peroxisome Proliferation Protein, Pex11
Source: Cells. 2022 Jan 4;11(1):157. doi: 10.3390/cells11010157 (PMC8750153; doi:10.3390/cells11010157)
Supplement: Supplementary file 1 [file cells-11-00157-s001.zip › cells-1500612-supplementary.pdf]

Supplementary Information Appendix

Supplementary Figures

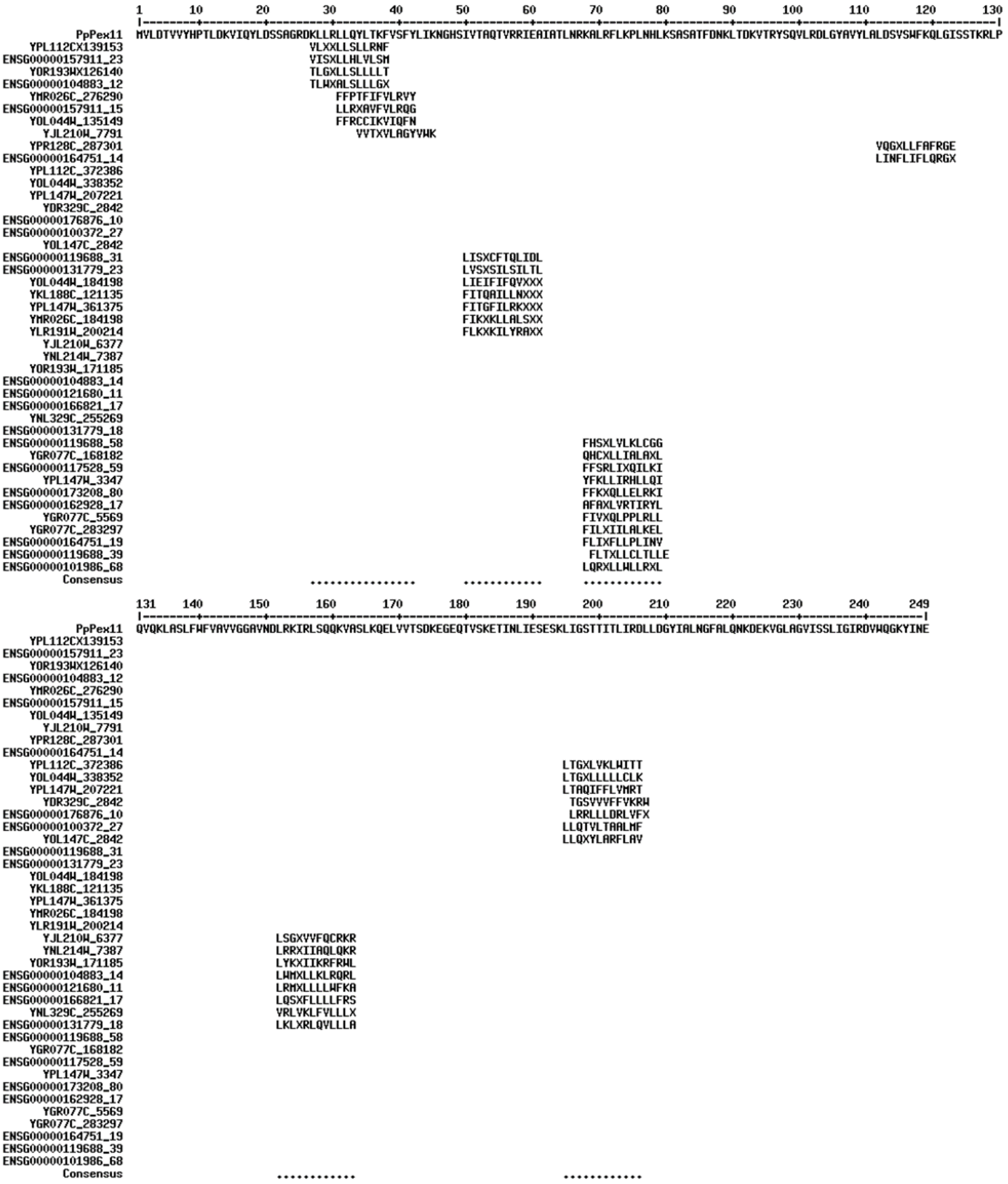

Figure S1. Alignment of Pex11 protein sequences with predicted Pex19-binding sites (Pex19-BS).

The *P. pastoris* Pex11 sequence was aligned by using MultAlin (http://multalin.toulouse.inra.fr/multalin[1]) with Pex19BS BLOCK prediction matrix containing both yeast and human Pex19 targeting elements[2, 3]. The Pex19BS BLOCK used in this study was from http://216.92.14.62/Target_signal.php.

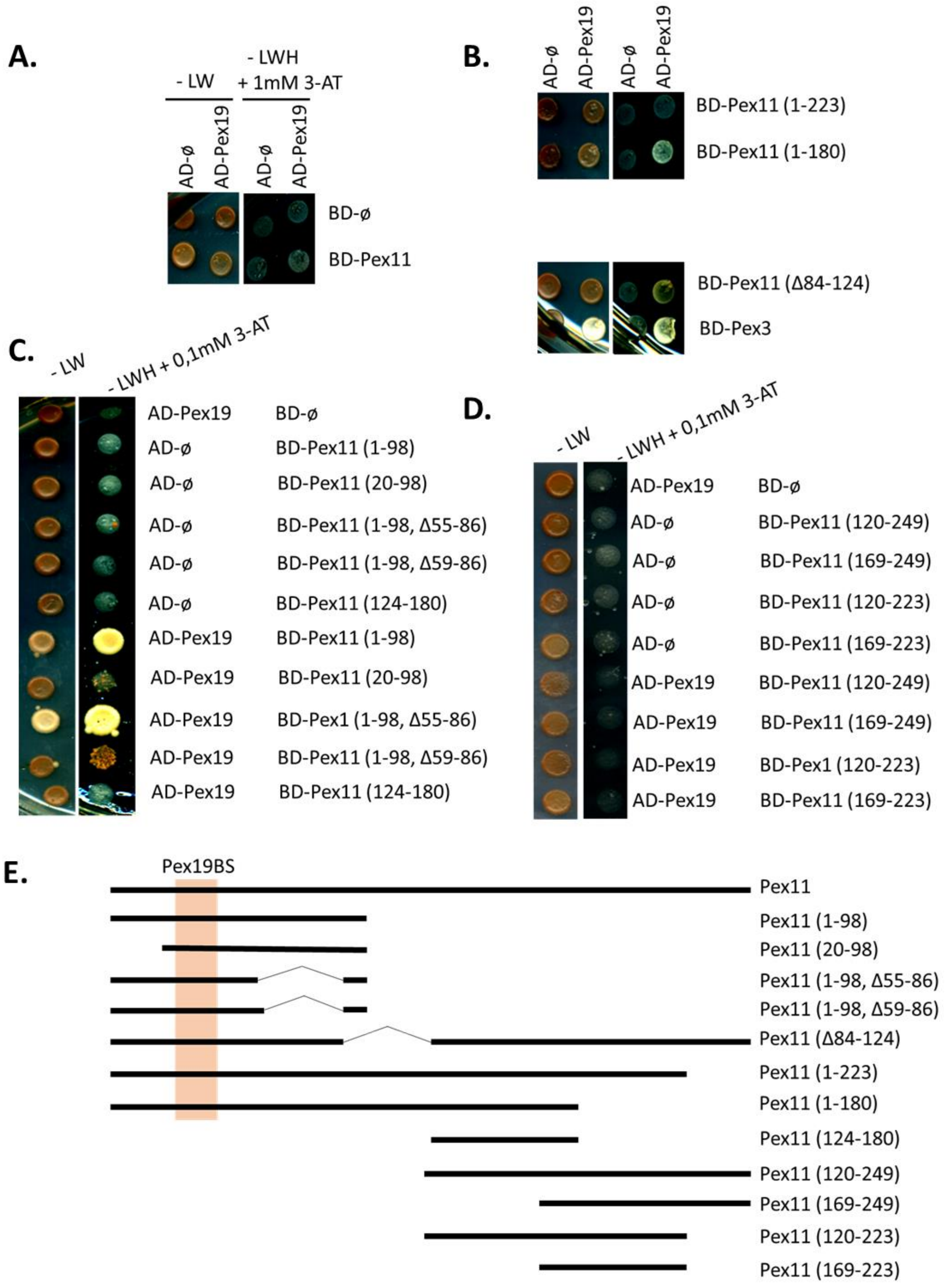

Figure S2. Pex11 interacts with Pex19 via its Pex19-BS.

A-D. Determination of the Pex19-BS module in PpPex11 by Y2H. Full-length PpPex11 and its various truncated forms were fused with the BD domain of GAL4 and evaluated for their ability to interact with AD-Pex19. The interaction between AD-

PpPex19 and BD-PpPex3 was used as a positive control. **E.** Schematic representation of Pex11 truncated forms used in this study is shown and the H2 helix (aa25-45), within which the Pex19-BS was mapped for other yeast Pex11s, is highlighted in orange. 3-AT, 3-amino-1,2,4-triazole; AD, activation domain; BD, DNA binding domain; -LW, yeast synthetic drop-out medium without leucine and tryptophan serving as a positive control to show equal plating of cells; -LWH, yeast synthetic drop-out medium without leucine, tryptophan and histidine.

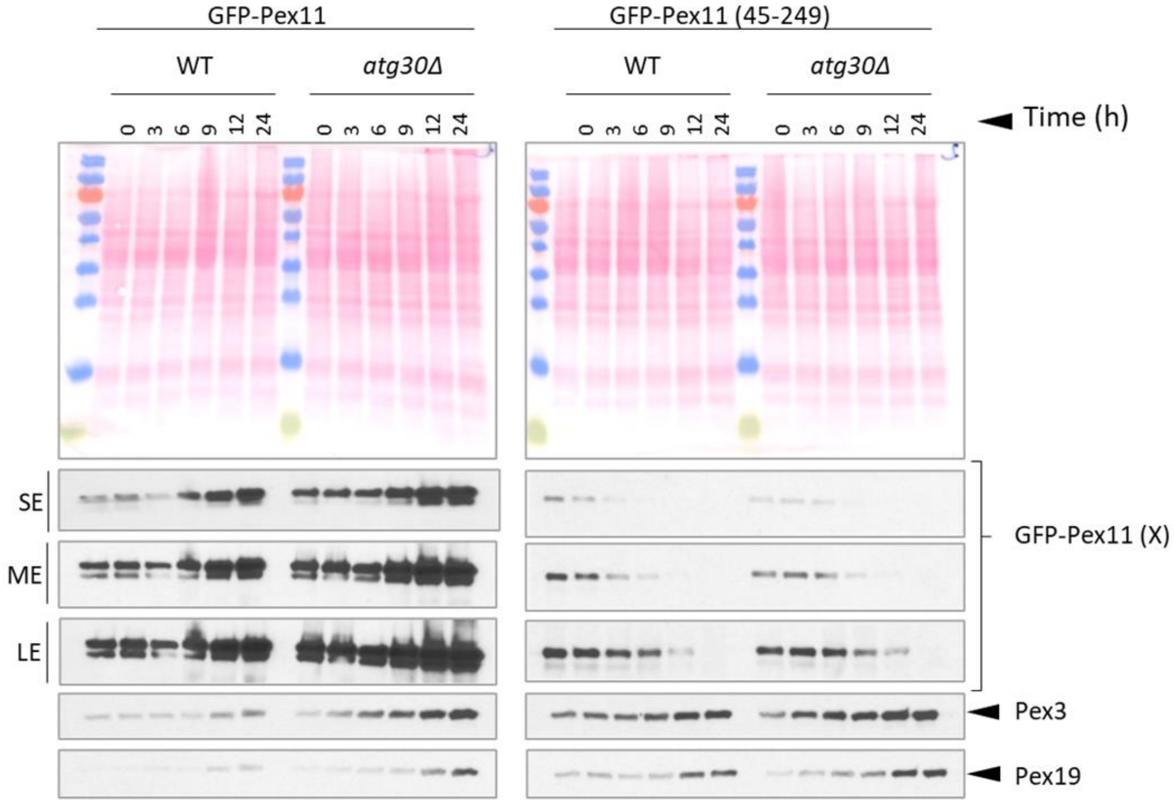

Figure S3. Pex11 (45-249) is not subject to Atg30-dependent autophagic degradation.

Western blot analysis of GFP-Pex11 and GFP-Pex11 (45-249) protein levels in WT and *atg30*Δ cells after methanol induction. WT and *atg30*Δ cells expressing GFP-Pex11 or GFP-Pex11 (45-249) (collectively labeled GFP-Pex11(X)) and expressed from the *PEX11* promoter were grown in methanol medium and 2 OD cells were collected at indicated time points. GFP-Pex11, Pex3 and Pex19 levels were visualized by specific antibodies, respectively. SE-short exposure, ME-moderate exposure and LE-long exposure.

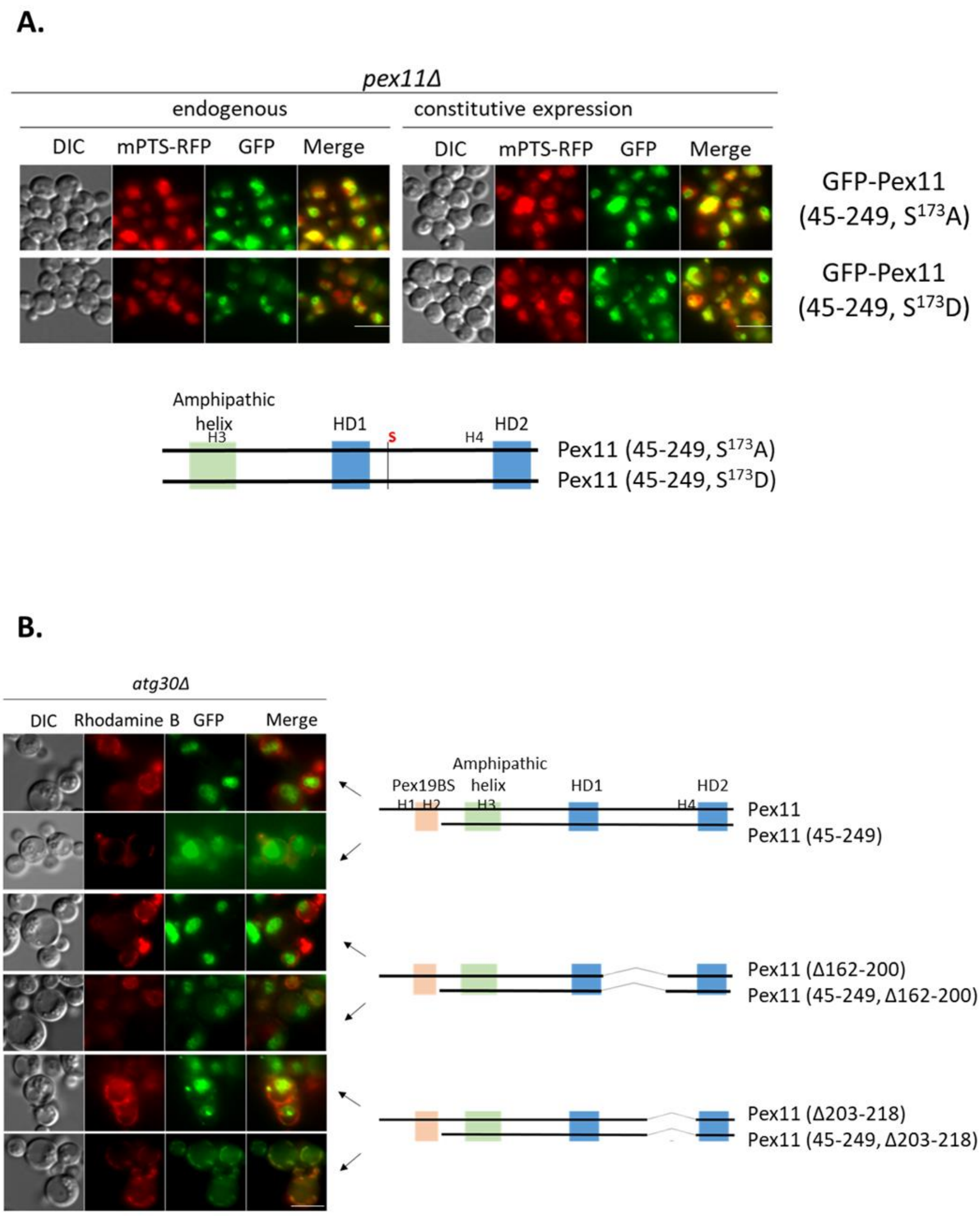

Figure S4. Mapping the novel, Pex19-independent mPTS in Pex11.

A. Pex11 (45-249) trafficking to peroxisomes does not depend on phosphorylation mediating the Pex11-Fis1 interaction. Fluorescence microscopy images of *pex11*Δ cells with mPTS-RFP for peroxisome visualization and expressing two Pex11 (45-249) mutants - namely GFP-Pex11(45-249, S173A) (constitutively-unphosphorylated; S173A)

and GFP-Pex11 (45-249, S173D) (constitutively-phosphorylated; S173D) were taken after 5 h of methanol induction. Bar= 5μm. Schematic representation of Pex11(45-249, S173A) and Pex11(45-249, S173D) truncated forms used in this study is shown on left. **B.** Fluorescence microscopy images of pexophagy-deficient *atg30*Δ cells expressing various truncated forms of GFP-Pex11 fusion proteins after 5 h in methanol medium. Cells were stained by Rhodamine B to label mitochondria prior to observation. Bar= 5μm. Schematic representation of Pex11 truncated forms used in this study is shown on the right.

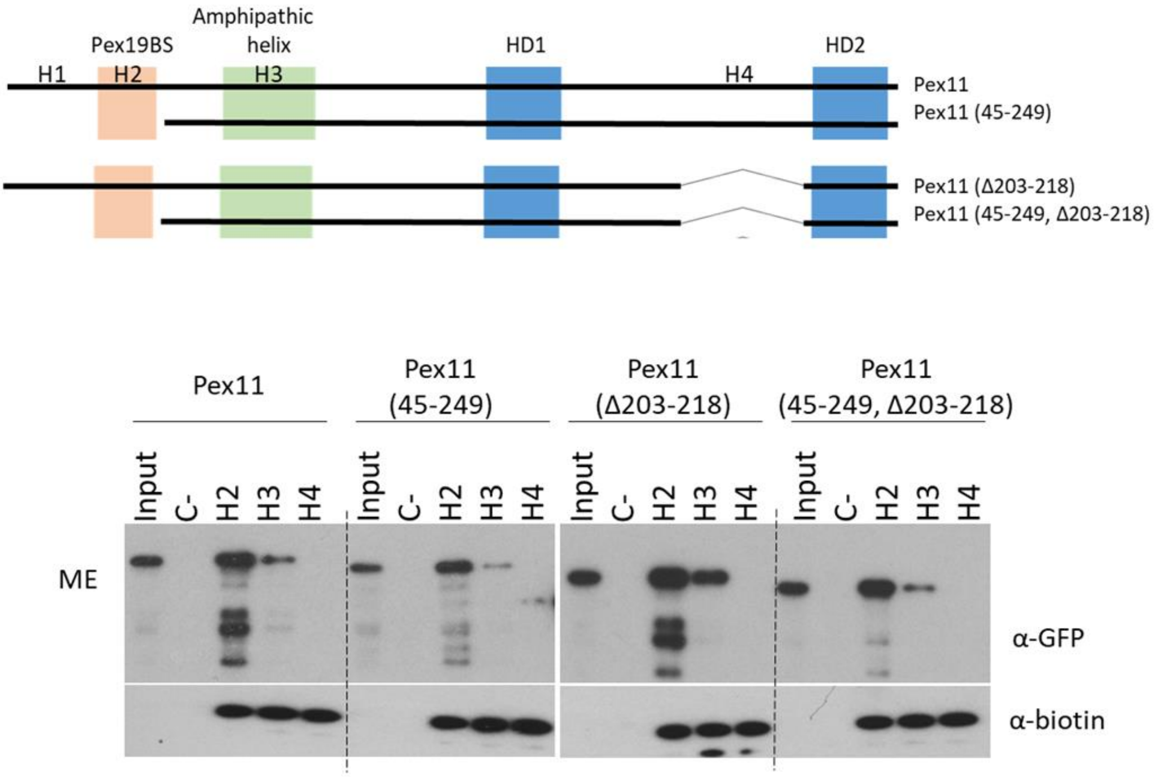

Figure S5. Novel mPTS located within helix H4 is not involved in Pex11 self-interaction.

Pull-down assay using biotinylated peptides corresponding to helices H2, H3 or H4 (sequences indicated in the Fig. 5) bound to streptavidin-coated resin as a bait and His_6-GFP-Pex11 or truncated forms (His_6-GFP-Pex11 (X)) used as a prey. Resins were washed, and proteins were eluted and analyzed by SDS-PAGE. His_6-GFP-Pex11 (X) fusion proteins were detected by immunoblotting with anti-GFP antibodies and the presence of peptides on the resin was verified by HRP-conjugated streptavidin. Shown is 30% of the input. ME, moderate exposure.

Supplementary Tables

Table S1. Plasmids used in this study.

S. cerevisiae vectors for Y2H					
Name	Background	Insert	Fusion protein	Selection	Reference
pGAD-GH	pGAD-GH	none	AD	Amp/Leu	TaKaRa
pGADT7	pGADT7	none	AD	Amp/Leu	TaKaRa
pGBKT7	pGBKT7	none	BD	Kan/Trp	TaKaRa
AD-Pex19	pGAD-GH	PpPex19	AD	Amp/Leu	(7)[4]
pKSN65	pGBKT7	PpPex3	BD	Kan/Trp	(8)[5]
pNR13	pGBT9	PpPex11	BD	Kan/Trp	(7)[4]
pKZR061	pGBKT7	PpPex11 (1-249)	BD	Kan/Trp	this study
pKZR062	pGBKT7	PpPex11 (1-223)	BD	Kan/Trp	this study
pKZR063	pGBKT7	PpPex11 (1-180)	BD	Kan/Trp	this study
pKZR088	pGBKT7	PpPex11 (1-180, Δ30-45)	BD	Kan/Trp	this study
pKZR089	pGBKT7	PpPex11 (1-180, L35P)	BD	Kan/Trp	this study
pKZR066	pGBKT7	PpPex11 (Δ84-124)	BD	Kan/Trp	this study
pKZR090	pGBKT7	PpPex11 (Δ84-124, Δ30-45)	BD	Kan/Trp	this study
pKZR091	pGBKT7	PpPex11 (Δ84-124, L35P)	BD	Kan/Trp	this study
pKZR067	pGBKT7	PpPex11 (1-98)	BD	Kan/Trp	this study
pKZR068	pGBKT7	PpPex11 (20-98)	BD	Kan/Trp	this study
pKZR069	pGBKT7	PpPex11 (1-98, Δ55-86)	BD	Kan/Trp	this study
pKZR070	pGBKT7	PpPex11 (1-98, Δ59-86)	BD	Kan/Trp	this study
pKZR071	pGBKT7	PpPex11 (124-180)	BD	Kan/Trp	this study
pKZR072	pGBKT7	PpPex11 (120-249)	BD	Kan/Trp	this study
pKZR073	pGBKT7	PpPex11 (169-249)	BD	Kan/Trp	this study
pKZR074	pGBKT7	PpPex11 (120-223)	BD	Kan/Trp	this study
pKZR075	pGBKT7	PpPex11 (169-223)	BD	Kan/Trp	this study
pKZR076	pGADT7	PpPex11 (1-98)	AD	Amp/Leu	this study
pKZR077	pGADT7	PpPex11 (20-98)	AD	Amp/Leu	this study
pKZR078	pGADT7	PpPex11 (1-98, Δ30-45)	AD	Amp/Leu	this study
pKZR079	pGADT7	PpPex11 (1-98, L35P)	AD	Amp/Leu	this study
pKZR080	pGADT7	PpPex11 (1-98, Δ55-86)	AD	Amp/Leu	this study
pKZR081	pGADT7	PpPex11 (1-98, Δ59-86)	AD	Amp/Leu	this study
Protein expression					
Name	Background	Insert	N-terminal tag	Selection	Reference
pMP03	pGEXKG	PpPex19	GST	Amp	this study
pKZR082	pACYCDuet-1	GFP-PpPex11 (1-249)	HIS_6-GFP	Cm	this study
pKZR083	pACYCDuet-1	GFP-PpPex11 (45-249)	HIS_6-GFP	Cm	this study
pKZR084	pACYCDuet-1	GFP-PpPex11 (1-223)	HIS_6-GFP	Cm	this study
pKZR085	pACYCDuet-1	GFP-PpPex11 (1-180)	HIS_6-GFP	Cm	this study
pKZR086	pACYCDuet-1	GFP-PpPex11 (Δ203-218)	HIS_6-GFP	Cm	this study
pKZR087	pACYCDuet-1	GFP-PpPex11 (45-249, Δ203-218)	HIS_6-GFP	Cm	this study

Table S2. Strains used in this study.

Strain	Background strain	Genotype	Reference
PPY12		*his4, arg4*	(9)[6]
sMY341		*PPY12h pex11Δ::Zeocin^R (pMYΔ11') arg4::pKSN29 (P_{GAP}-mPTS-RFP, ARG4) his4*	(10)[7]
sKZR30	sMY341	*PPY12h pex11Δ::Zeocin^R (pMYΔ11') arg4::pKSN29 (P_{GAP}-mPTS-RFP, ARG4) his4::pMY57 (P_{PEX11}-GFP-PEX11, HIS4)*	this study
sKZR31	sMY341	*PPY12h pex11Δ::Zeocin^R (pMYΔ11') arg4::pKSN29 (P_{GAP}-mPTS-RFP, ARG4) his4::pKZR90 (P_{PEX11}-GFP-PEX11 (45-249), HIS4)*	this study
sKZR32	sMY341	*PPY12h pex11Δ::Zeocin^R (pMYΔ11') arg4::pKSN29 (P_{GAP}-mPTS-RFP, ARG4) his4::pMY59 (P_{PEX11}-PEX11-2HA, HIS4)*	this study
sKZR33	sMY341	*PPY12h pex11Δ::Zeocin^R (pMYΔ11') arg4::pKSN29 (P_{GAP}-mPTS-RFP, ARG4) his4::pKZR89 (P_{PEX11}-GFP-PEX11 (20-249), HIS4)*	this study
sKZR34	sMY341	*PPY12h pex11Δ::Zeocin^R (pMYΔ11') arg4::pKSN29 (P_{GAP}-mPTS-RFP, ARG4) his4::pKZR91 (P_{PEX11}-GFP-PEX11 (1-223), HIS4)*	this study
sKZR35	sMY341	*PPY12h pex11Δ::Zeocin^R (pMYΔ11') arg4::pKSN29 (P_{GAP}-mPTS-RFP, ARG4) his4::pKZR92 (P_{PEX11}-GFP-PEX11 (1-180), HIS4)*	this study
sKZR36	sMY341	*PPY12h pex11Δ::Zeocin^R (pMYΔ11') arg4::pKSN29 (P_{GAP}-mPTS-RFP, ARG4) his4::pKZR100 (P_{PEX11}-GFP-PEX11 (45-223), HIS4)*	this study
sKZR37	sMY341	*PPY12h pex11Δ::Zeocin^R (pMYΔ11') arg4::pKSN29 (P_{GAP}-mPTS-RFP, ARG4) his4::pKZR101 (P_{PEX11}-GFP-PEX11 (156-249), HIS4)*	this study
sKZR38	sMY341	*PPY12h pex11Δ::Zeocin^R (pMYΔ11') arg4::pKSN29 (P_{GAP}-mPTS-RFP, ARG4) his4::pKZR102 (P_{PEX11}-GFP-PEX11 (45-249, S173A), HIS4)*	this study
sKZR39	sMY341	*PPY12h pex11Δ::Zeocin^R (pMYΔ11') arg4::pKSN29 (P_{GAP}-mPTS-RFP, ARG4) his4::pKZR103 (P_{PEX11}-GFP-PEX11 (45-249, S173D), HIS4)*	this study
sKZR40	sMY341	*PPY12h pex11Δ::Zeocin^R (pMYΔ11') arg4::pKSN29 (P_{GAP}-mPTS-RFP, ARG4) his4::pKZR104 (P_{GAP}-GFP-PEX11 (45-249, S173A), HIS4)*	this study

sKZR41	sMY341	*PPY12h pex11Δ::Zeocin^R (pMYΔ11') arg4::pKSN29 (P_{GAP}-mPTS-RFP, ARG4) his4::pKZR105 (P_{GAP}-GFP-PEX11 (45-249, S173D), HIS4)*	this study
sKZR42	sMY341	*PPY12h pex11Δ::Zeocin^R (pMYΔ11') arg4::pKSN29 (P_{GAP}-mPTS-RFP, ARG4) his4::pKZR97 (P_{PEX11}-GFP-PEX11 (Δ203-218) HIS4)*	this study
sKZR43	sMY341	*PPY12h pex11Δ::Zeocin^R (pMYΔ11') arg4::pKSN29 (P_{GAP}-mPTS-RFP, ARG4) his4::pKZR99 (P_{PEX11}-GFP-PEX11 (45-249, Δ203-218), HIS4)*	this study
sKZR44	PPY12	*his4::pMY57 (P_{PEX11}-GFP-PEX11, HIS4), arg4*	this study
sKZR45	PPY12	*his4::pKZR90 (P_{PEX11}-GFP-PEX11 (45-249), HIS4), arg4*	this study
sJCF2347		*GS115 atg30Δ::Zeocin^R (pJCF56), his4*	(7)[4]
sKZR45	sJCF2347	*GS115 atg30Δ::Zeocin^R (pJCF56), his4::pKZR88 (P_{PEX11}-GFP, HIS4)*	this study
sKZR46	sJCF2347	*GS115 atg30Δ::Zeocin^R (pJCF56), his4::pMY57 (P_{PEX11}-GFP-PEX11, HIS4)*	this study
sKZR47	sJCF2347	*GS115 atg30Δ::Zeocin^R (pJCF56), his4::pKZR90 (P_{PEX11}-GFP-PEX11 (45-249), HIS4)*	this study
sKZR48	sJCF2347	*GS115 atg30Δ::Zeocin^R (pJCF56), his4::pKZR91 (P_{PEX11}-GFP-PEX11 (1-223), HIS4)*	this study
sKZR49	sJCF2347	*GS115 atg30Δ::Zeocin^R (pJCF56), his4::pKZR96 (P_{PEX11}-GFP-PEX11 (Δ162-200), HIS4)*	this study
sKZR50	sJCF2347	*GS115 atg30Δ::Zeocin^R (pJCF56), his4::pKZR98 (P_{PEX11}-GFP-PEX11 (45-249, Δ162-200), HIS4)*	this study
sKZR51	sJCF2347	*GS115 atg30Δ::Zeocin^R (pJCF56), his4::pKZR97 (P_{PEX11}-GFP-PEX11 (Δ203-218), HIS4)*	this study
sKZR52	sJCF2347	*GS115 atg30Δ::Zeocin^R (pJCF56), his4::pKZR99 (P_{PEX11}-GFP-PEX11 (45-249, Δ203-218), HIS4)*	this study
sSSM46	sJCF2347	*GS115 atg30Δ::Zeocin^R (pJCF56), his4::pMY59 (P_{PEX11}-PEX11-2HA, HIS4)*	this study
sSSM48	SJCF2435	*PPY12 atg30Δ::Zeocin^R (pJCF56), pex3Δ::ARG4, his4::pMY59 (P_{PEX11}-PEX11-2HA, HIS4)*	this study
sSSM49	SJCF2436	*PPY12 atg30Δ::G418^R (pJCF421), pex19Δ::Zeocin^R, his4::pMY59 (P_{PEX11}-PEX11-2HA, HIS4)*	this study
sJCF1403	GS115	*GS115 arg4::pJCF523 (P_{TOM20}-TOM20-mCherry, HYGROMYCIN^R), his4*	(11) [8]
sKZR52	sJCF1403	*GS115 arg4::pJCF523 (P_{TOM20}-TOM20-mCherry, HYGROMYCIN^R), his4::pMY57 (P_{PEX11}-GFP-PEX11, HIS4)*	this study

sKZR53	sJCF1403	*GS115 arg4::pJCF523 (P_{TOM20}-TOM20-mCherry, HYGROMYCIN^{R}), his4::pKZR90 (P_{PEX11}-GFP-PEX11 (45-249), HIS4)*	this study
sKZR54	sJCF1403	*GS115 arg4::pJCF523 (P_{TOM20}-TOM20-mCherry, HYGROMYCIN^{R}), his4::pMY54 (P_{GAP}-GFP-PEX11, HIS4)*	this study
sJCF2286	sKF13	*GS115 pex19Δ::ZEOCIN^{R}, arg4::pJCF523 (P_{TOM20}-TOM20-mCherry, HYGROMYCIN^{R}), his4*	this study
sKZR54	sJCF2286	*GS115 pex19Δ::ZEOCIN^{R}, arg4::pJCF523 (P_{TOM20}-TOM20-mCherry, HYGROMYCIN^{R}), his4::pMY57 (P_{PEX11}-GFP-PEX11, HIS4)*	this study
sKZR55	sJCF2286	*GS115 pex19Δ::ZEOCIN^{R}, arg4::pJCF523 (P_{TOM20}-TOM20-mCherry, HYGROMYCIN^{R}), his4::pKZR90 (P_{PEX11}-GFP-PEX11 (45-249), HIS4)*	this study
sKZR56	sJCF2286	*GS115 pex19Δ::ZEOCIN^{R}, arg4::pJCF523 (P_{TOM20}-TOM20-mCherry, HYGROMYCIN^{R}), his4::pMY54 (P_{GAP}-GFP-PEX11, HIS4)*	this study

Table S3. Peptides used in this study.

Name	Sequence	N-terminal modification	Purity	Reference
1	**KLLRLLQYLTKFVS**	none	crude	this study
2	**RLLQYLTKFVSFYLIK**	none	crude	this study
3	**LIGSTTITLIRDLLD**	none	crude	this study
4	**TTITLIRDLLDGYI**	none	crude	this study
5	**TLIRDLLDGYIALN**	none	crude	this study
6	**DLLDGYIALNGFALQ**	none	crude	this study
7	**DGYIALNGFALQNKD**	none	crude	this study
H2	**RDKLLRLLQYLTKFVSFYLIK**	Biotin-Ahx	crude	this study
H3	**IEAIATLNRKALRFLKPLNHL**	Biotin-Ahx	crude	this study
H4	**TLIRDLLDGYIALNGFALQ**	Biotin-Ahx	crude	this study
H2 PEX11β	**RERLCRAAQYACSLLGHALQR**	Biotin-Ahx	crude	this study
H2 R1N	**NDKLLRLLQYLTKFVSFYLIK**	Biotin-Ahx	crude	this study
H2 K3N	**RDNLLRLLQYLTKFVSFYLIK**	Biotin-Ahx	crude	this study
H2 L4K	**RDKKLRLLQYLTKFVSFYLIK**	Biotin-Ahx	crude	this study
H2 R6N	**RDKLLNLLQYLTKFVSFYLIK**	Biotin-Ahx	crude	this study
H2 L7A	**RDKLLRALQYLTKFVSFYLIK**	Biotin-Ahx	crude	this study
H2 L8A	**RDKLLRLAQYLTKFVSFYLIK**	Biotin-Ahx	crude	this study
H2 Y10A	**RDKLLRLLQALTKFVSFYLIK**	Biotin-Ahx	crude	this study
H2 L8A, Y10A	**RDKLLRLAQALTKFVSFYLIK**	Biotin-Ahx	crude	this study
H2 L11A	**RDKLLRLLQYATKFVSFYLIK**	Biotin-Ahx	crude	this study
H2 muts	**RDKLLRAAQYATKFVSFALIK**	Biotin-Ahx	crude	this study
H2 K13N	**RDKLLRLLQYLTNFVSFYLIK**	Biotin-Ahx	crude	this study
H2 Y18A	**RDKLLRLLQYLTKFVSFALIK**	Biotin-Ahx	crude	this study
H2 Y18K	**RDKLLRLLQYLTKFVSFKLIK**	Biotin-Ahx	crude	this study
H2 L4KY18K	**RDKKLRLLQYLTKFVSFKLIK**	Biotin-Ahx	crude	this study

Supplementary references

1. Corpet F (1988) Multiple sequence alignment with hierarchical clustering. *Nucleic Acids Res* 16(22):10881-10890.
2. Rottensteiner H, *et al.* (2004) Peroxisomal membrane proteins contain common Pex19p-binding sites that are an integral part of their targeting signals. *Mol Biol Cell* 15(7):3406-3417.
3. Halbach A, *et al.* (2005) Function of the PEX19-binding site of human adrenoleukodystrophy protein as targeting motif in man and yeast. PMP targeting is evolutionarily conserved. *J Biol Chem* 280(22):21176-21182.
4. Farre JC, *et al.* (2017) A New Yeast Peroxin, Pex36, a functional homolog of mammalian PEX16, functions in the ER-to-Peroxisome traffic of peroxisomal membrane proteins. *J Mol Biol* 429(23):3743-3762.

5. Burnett SF, Farre JC, Nazarko TY, & Subramani S (2015) Peroxisomal Pex3 activates selective autophagy of peroxisomes via interaction with the pexophagy receptor Atg30. *J Biol Chem* 290(13):8623-8631.
6. Gould SJ, McCollum D, Spong AP, Heyman JA, & Subramani S (1992) Development of the yeast *Pichia pastoris* as a model organism for a genetic and molecular analysis of peroxisome assembly. *Yeast* 8(8):613-628.
7. Joshi S, Agrawal G, & Subramani S (2012) Phosphorylation-dependent Pex11p and Fis1p interaction regulates peroxisome division. *Mol Biol Cell* 23(7):1307-1315.
8. Farre JC, Burkenroad A, Burnett SF, & Subramani S (2013) Phosphorylation of mitophagy and pexophagy receptors coordinates their interaction with Atg8 and Atg11. *EMBO Rep* 14(5):441-449.
